# A Multicenter Study of Multimorbidity in Older Adult Inpatients in China

**DOI:** 10.1007/s12603-020-1311-x

**Published:** 2020-01-10

**Authors:** L. Zhang, L. Ma, F. Sun, Zhe Tang, Piu Chan

**Affiliations:** 1 Department of Geriatrics, Xuanwu Hospital, Capital Medical University, Beijing, 100053 China; 2 Beijing Geriatric Healthcare Center, Xuanwu Hospital, Capital Medical University, Beijing, China; 3 Parkinson Disease Center of Beijing Institute for Brain Disorders, Key Laboratory for Neurodegenerative Disease of the Ministry of Education, Beijing Key Laboratory for Parkinson’s Disease, Beijing, China; 4 Department of Neurobiology, and Neurology, Xuanwu Hospital of Capital Medical University, Beijing Institute of Geriatrics, Beijing, China; 5 Department of Neurobiology, Xuanwu Hospital of Capital Medical University, Beijing, 100053 China; 6 Beijing Geriatric Healthcare Center, Xuanwu Hospital, Capital Medical University, Beijing, 100053 China

**Keywords:** Multimorbidity, morbidity, older adults, inpatients, disability

## Abstract

**Objectives:**

Multimorbidity is common in older hospitalized adults. To date, however, few studies have addressed multimorbidity in the older population of Chinese inpatients. We aimed to investigate the multimorbidity rate and associated risk factors in older adult inpatients in China.

**Design, Setting, Participants:**

This study was conducted in the medical wards of a tertiary-care hospital from. The patients were recruited aged between 60 to 101 (74.14±8.46) years.

**Measurements:**

Data were obtained from the China Comprehensive Geriatric Assessment Study, conducted in 2011–2012 in China. A total of 4,633 inpatients older than 60 years was recruited from 12 hospitals in 7 cities throughout China. The prevalence of comorbidity, distribution of common chronic diseases, and the associated risk factors were studied.

**Results:**

A total of 4,348 people aged 60 to 101 (74.14±8.46) years completed questionnaires. The average frequency of multimorbidity was 69.3% (95% CI, 67.9% to 70.6%). The prevalence of multimorbidity increased with age and was higher in men (71.6%; 95% CI, 69.9% to 73.3%) than in women (65.3%, 95% CI 63.0% to 67.6%), and higher in the northern region (71.7%, 95% CI 69.9% to 73.5%) than in the southern region (66.0%; 95% CI, 63.8% to 68.1%). The most frequent chronic diseases were hypertension, coronary heart disease, diabetes, cataract, and stroke. Area (OR=0.556; 95% CI, 0.465 to 0.666), region (OR=0.834; 95% CI, 0.723 to 0.962), body mass index (BMI) (OR=1.124; 95% CI, 1.017 to 1.242), and impairment of activities of daily living (OR=0.911; 95% CI, 0.855 to 0.970) were independent factors associated with multimorbidity.

**Conclusions:**

Multimorbidity is common in older Chinese inpatients with a national prevalence of 69.3% that increases in line with age. Age, region, area, BMI, and daily activities were independent factors significantly associated with multimorbidity in older inpatients. Clinicians should therefore focus more attention on multimorbidity.

## Introduction

Multimorbidity, defined as the co-occurrence of 2 or more chronic conditions, is very common among older adults (1). With a continuing increase in life expectancy, multimorbidity has become a worldwide public health issue and is associated with increasing adverse health outcomes, such as mortality, disability, poor quality of life, hospitalizations, and concomitant use of healthcare resources and expenditure (2–4). A systematic review of 41 articles showed that the prevalence of multimorbidity from different countries lies between 20% and 30% for the entire population and 55% to 98% for individuals older than 65 years (5). Multimorbidity increases in parallel with both social deprivation and age, with almost a quarter of the United Kingdom population as a whole and two-thirds of people aged 65 years or over affected (6). In the United States, almost 3 out of 4 older adults reportedly have multiple chronic conditions (7): in 2000, 57 million Americans had multiple chronic conditions and by 2020 this number is expected to grow to 81 million (8). Approximately 65% of patients aged 75 or older living in Tokyo who used medical care had 3 or more co-occurring diseases (9), while in Canada, of the 18% of the total population considered multimorbid, 57% were older than 65 years (10).

Research on multimorbidity of older adults in China is still at the early stage. For the community-dwelling older population, a cross-sectional community health survey in southern China showed that the average number of chronic conditions was 1.68 ±1.60, and the prevalence of multimorbidity was reported to be 45.5% (11). Meanwhile, a study from northeastern China reported that almost a quarter (24.7%) of older adults was found to be multimorbid for chronic diseases (12). In another study from Nanjing, the prevalence of multimorbidity was 49.4% in community-dwelling older adults in urban areas (13). A large cross-sectional survey among 162,464 community household residents in southern China reported that more than 1 in 10 of the total study population (11.1%, 95% confidence interval [CI] 10.6 to 11.6) had 2 or more chronic conditions from a list of 40 morbidities (14). There are few studies of multimorbidity in hospitalized older adult patients in China. A survey of discharged patients in Hong Kong showed that 33.9% had 2 or more comorbid conditions (15). In China, which maintains a large population of older people with multimorbidity, most clinical guidelines nonetheless focus almost exclusively on single conditions (16), resulting in numerous hospital visits, polypharmacy, repeated investigations, and substantial treatment burden (16,17). A better understanding of the epidemiology of multimorbidity is necessary in order to develop interventions, reduce the healthcare burden, and align healthcare services more closely with patients’ needs. Multimorbidity is of particular relevance to geriatricians because the number of morbidities and proportion of the population with multimorbidity increases substantially with age (6). The US Department of Health and Human Services has emphasized the importance of identifying common patterns in the occurrence of 2 or 3 diseases to guide the development of specific interventions for drug interactions (8). In January 2019, the Guiding Principles on the care of older adults with multimorbidity were released (18). However, there is a lack of large-scale epidemiological studies of the incidence of multimorbidity among older inpatients across China. This study aimed to investigate the prevalence and risk factors of multimorbidity in this population.

## Methods

### Participants

For this retrospective study, inpatient data were obtained from the China Comprehensive Geriatric Assessment Study (CCGAS) from January 2011 to December 2012 (19). According to the division of north and south China by Qinling mountains/Huaihe river as boundary, 12 tertiary hospitals from 7 provinces representative of the north and south of China were chosen (north including Beijing, Harbin, and Xi’an; south including Changsha, Chongqing, Chengdu, and Shanghai). A sample was chosen randomly from hospitalized patients aged 60 years and older. In total, 4,633 inpatients were included from the geriatric departments. All patients provided written informed consent.

### Data collection

Our survey included sociodemographic variables, social function (e.g., activities of daily living [ADL] and instrumental ADL [IADL]), anthropometric measurements, chronic disease history, life behavior and habits (smoking, alcohol consumption, physical exercise, eating habits, sleep, and participation in social activities), mental health assessment (the Geriatric Depression Scale), cognitive function assessment (Mini-Mental State Examination), and medical conditions.

All diseases were diagnosed based on criteria set by the International Classification of Diseases, 10th version (ICD-10), including hypertension, coronary heart disease, diabetes, chronic obstructive pulmonary disease, cataract, stroke, transient ischemic attack, osteoarthritis, hearing loss, dementia, cancer, chronic kidney disease, hepatitis, schizophrenia, and gastrointestinal disease.

### Definitions

Older adults who suffered 2 or more chronic diseases were defined as multimorbid. Assessment of disability used an internationally accepted instrument that measured capability in ADL to assess physical function. The instrument comprises ADL (bathing, dressing, toileting, indoor walking, getting in/out of bed, and eating) and IADL (shopping, phone calls, housekeeping, laundry, money management, medication, and transportation). Thus, for the purposes of this study, those with one or more impaired ADL or IADL functions were defined as disabled (20).

### Statistical methods

The database established by Epi-Data was exported into SPSS19.0 after verification and confirmation of statistical analyses. The occurrences of multimorbidity were counted by sex, area, and age group. Chi-squared tests were used to compare the prevalence of the 15 diseases among sex and age groups. The sex-specific and area-specific detection rates were determined as the ratio of the number in the corresponding age group. Measurement data were compared by t tests, and rates were compared by chi-squared tests. Logistic analysis was carried out to explore the association of potential risk factors with multimorbidity. The results are presented as odds ratio (OR) and 95% CI.

## Results

Demographic characteristics of morbidity in older inpatients Of 4,633 inpatients, 285 (6.15%) subjects were excluded because of incomplete or inconsistent data. A total of 4,348 subjects were finally included in the analysis, whose age ranged from 60 to 101 (73.56±8.38) years. There were 375 (8.6%) subjects without chronic diseases, 962 (22.1%) with 1 disease, and 3,031 (69.3%) with 2 or more diseases. As expected, multimorbidity increased with aging and reached the highest frequency of 81.6% for people older than 80 years (Table 1).

**Table 1:** Demographic characteristics and morbidity of multimorbidity of respondents

	**Characteristic**	**Morbidity**			**χ2 test**	
		**Total**	**Number**	**Rate(%)(95% CI)**	**Chi-Square**	**P-Value**
Total		4348	3031	69.3(67.9–70.6)		
Age (year)	60–64	777	397	51.1(47.6–54.6)	254.657	<0.001
	65–69	766	464	60.6(57.1–64.0)		
	70–74	804	564	70.1(667.0–73.3)		
	75–79	804	609	75.7(72.8–78.7)		
	80+	1197	977	81.6(79.4–83.8)		
Region	North	2497	1790	71.7(69.9–73.5)	16.343	<0.001
	South	1851	1221	66.0(63.8–68.1)		
Area	Urban	3391	2495	73.6(72.1–75.1)	135.454	<0.001
	Rural	957	516	53.9(50.8–57.1)		
Sex	Male	2712	1943	71.6(69.9–73.3)	19.404	<0.001
	Female	1636	1068	65.3(63.0–67.6)		
Marital status	Married/cohabit	3603	2484	67.9(65.9–68.9)	26.176	<0.001
	Widow/separated	689	527	76.5(73.3–79.7)		
Educational level	Lower than middle school	1044	686	65.7(62.8–68.6)	29.548	<0.001
	Middle school	882	579	65.6(62.5–68.8)		
	High school	891	610	68.5(65.4–71.5)		
	College and above	1531	1136	74.2(72.0–76.4)		
BMI (kg/m2)	<18.5	216	156	72.2(66.2–78.2)	23.882	<0.001
	18.5–23.9	1857	1216	65.5(63.3–67.6)		
	24–27.9	1807	1288	71.3(69.2–73.4)		
	≥28	467	350	74.9(71.0–78.9)		
Smoking	Yes	1495	1073	71.8(69.5–74.1)	6.807	0.009
	No	2853	1938	67.9(66.2–69.6)		
Alcohol Drinking	Yes	1024	746	72.9(70.1–75.6)	8.158	0.004
	No	3324	2265	68.1(66.6–69.7)		
Daily exercise time (per day)	No exercise	1147	830	72.4(69.8–75.0)		
	Half a hour	1055	748	70.9(68.2–73.6)	14.019	0.007
	1 hour	1400	946	67.6(65.1–70.0)		
	2–3 hours	643	421	65.5(61.8–69.2)		
	3 hours	103	66	64.1(54.7–73.5)		

Compared to different sex, area, region, age group, marital status, educational level, BMI, smoking, alcohol drinking, and daily exercise time. p<0.05 was statistically significant within the group; *Northern cities included Beijing, Xi’an, and Harbin, and southern cities included Chengdu, Chongqing, Changsha, and Shanghai.

### Risk factors associated with multimorbidity

We further investigated the factors that might influence the prevalence of multimorbidity (Table 1). There was a significant difference in the distribution of multimorbidity between north and south (71.1% vs 66.0%, p<0.001), men and women (71.6% vs 65.3%, p<0.001), urban and rural areas (73.6% vs 53.9%, p<0.001), and married/cohabiting and widowed/separated (67.9% vs 76.5%, p<0.001). People with normal body mass index (BMI) (18.5 ≤ BMI < 23.9, 65.5%) had lower multimorbidity than those with abnormal BMI (BMI < 18.5, 72.2%; 24 ≤ BMI < 27.9, 71.2%; BMI ≤ 28, 74.9%). In addition, higher prevalence of multimorbidity was associated with smoking (p=0.009) and drinking alcohol (p=0.004). There was also a significant difference in education level (lower than middle school, 65.7%; middle school, 65.6%; high school, 68.5%; college and above, 74.2%; p<0.001) and daily exercise time (less than half an hour, 72.4%; half an hour to 1 hour, 70.9%; 1 hour, 67.6%; 2–3 hours, 65.5%; >3 hours, 64.1%; p<0.001).

Results of the forward stepwise logistic regression analysis for risk factors associated with multimorbidity are shown in Table 2. After adjusting for age, region, area, gender, and other risk factors, age (adjusted OR=1.359, 95% CI 1.291 to 1.430), region (adjusted OR=0.834, 95% CI 0.723 to 0.962), area (adjusted OR=0.556, 95% CI 0.465 to 0.666), BMI (adjusted OR=1.185, 95% CI 1.080 to 1.299), and daily activity time (adjusted OR=0.911, 95% CI 0.855 to 0.970) were independent factors significantly associated with multimorbidity in older adult inpatients.

**Table 2:** Logistic regression models analyzing the relationship between multimorbidity and sample characteristics

**Factor**	**Unadjusted OR (95%CI)**	**p-Value**	**Adjusted OR (95%CI)**	**p-Value**
Age	1.435(1.370–502)	<0.001	1.359(1.291–1.430)	<0.001
Region	0.765(0.672–0.872)	<0.001	0.834(0.723–0.962)	0.013
Area	0.420(0.362–0.487)	<0.001	0.556(0.465–0.666)	<0.001
Sex(female)	0.744(0.652–0.849)	<0.001	0.886(0.751–1.045)	0.151
Marital status(married)	1.529(1.273–1.859)	<0.001	1.155(0.939–1.422)	0.172
Education level(illiteracy)	1.134(1.079–1.192)	<0.001	1.035(1.969–1.2037	0.305
BMI	1.233(1.129–1.347)	<0.001	1.185(1.080–1.299)	<0.001
Income(<2000yuan)	1.377(1.263–1.501)	<0.001	1.011(0.905–1.129)	0.849
Drinking	1.255(1.074–1.466)	0.004	1.184(0.984–1.426)	0.074
Smoking	1.200(1.046–1.377)	0.009	1.070(0.899–1.274)	0.448
Activity	0.896(0.845–0.950)	<0.001	0.911(0.855–0.970)	0.004

Multimorbidity was taken as the dependent variable (1 = yes;0 = none); age (60–64 years old), region (north), area (urban), gender (female), marital status (married), education level (illiteracy), BMI (<18), income (<2,000 RMB/year), drinking (no), smoking (no), and daily activity time (less than 30 min/day) were taken as corresponding variables.

### Burden of multimorbidity by sex, region, and age

The burden of multimorbidity is represented by the number of chronic diseases in each individual. Table 3 shows the effects of age, gender, and region on the burden of multimorbidity of chronic diseases. There were 375 (8.6%) people without chronic conditions, while 2 (22.1%), 3 (25.1%), and 4 diseases (18.8%) were the most frequent conditions among the cohort. The proportion of men with more than 2 chronic diseases was higher than that of women, especially in those with more than 5 chronic diseases (8.4% vs 3.9%), while the number of inpatients in the north with more than 3 chronic diseases was higher than that in the south. The number of chronic diseases increased with age, especially in those with more than 5 diseases. The age ranging from 60 to 69 had the highest rate of 2 chronic diseases (30.7%). The proportion of older inpatients suffering from 3 chronic diseases was the highest (29.4%) in the 70- to 79-year-old age group, and with 4 chronic diseases (19.7%) in the inpatients older than 80.

**Table 3:** Prevalence of different numbers of chronic diseases

	**Total number**	**no disease**	**1 chronic disease**	**2 chronic diseases**	**3 chronic diseases**	**4 chronic diseases**	**5 chronic diseases**	**>5 chronic diseases**
Total	4348	375(8.6%)	962(22.1%)	1092(25.1%)	817(18.8%)	518(11.9%)	293(6.7%)	291(6.7%)
Sex								
Male	2712	221(8.1%)	548(20.2%)	652(24.0%)	522(19.2%)	335(12.4%)	206(7.6%)	228(8.4%)
Female	1636	154(9.4%)	414(25.3%)	440(26.9%)	295(18.0%)	183(11.2%)	87(5.3%)	63(3.9%)
Region								
North	2497	215(8.6%)	492(19.7%)	592(23.7%)	456(18.3%)	325(13.0%)	211(8.5%)	206(8.2%)
South	1851	160(8.6%)	470(25.4%)	500(27.0%)	361(19.5%)	193(10.4%)	82(4.4%)	85(4.6%)
Age (years)								
60–69	1543	209(13.5%)	473(30.7%)	454(29.4%)	257(16.7%)	96(6.2%)	36(2.3%)	18(1.2%)
70–79	1608	114(7.1%)	321(20.0%)	447(27.8%)	324(20.1%)	208(12.9%)	110(6.8%)	84(5.2%)
80+	1197	52(4.3%)	168(14.0%)	191(16.0%)	236(19.7%)	214(17.9%)	147(12.3%)	189(15.8%)

### Rank prevalence of chronic diseases

**Figure 1:**
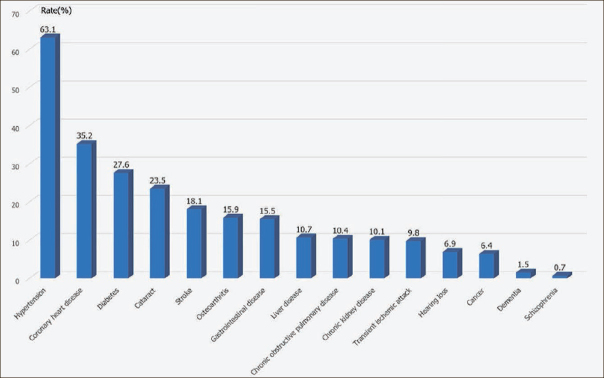
Ranking of prevalence of the 15 chronic diseases in older adult inpatients

Hypertension was ranked the most frequent chronic disease, with prevalence as high as 63.06%, far higher than for other chronic disease. Prevalence of other chronic diseases followed the order coronary heart disease (35.17%), diabetes (27.62%), cataract (23.51%), stroke (18.08%), osteoarthritis (15.92%), and others (Figure 1).

### Relationship between disability and multimorbidity

When disability was defined as having one or more ADL and IADL impairment, 673 (15.5%) participants had concomitant multimorbidity and disability, 1,193 (27.4%) had neither disability nor multimorbidity, 144 (3.3%) had only disability, and 2,338 (53.8%) had only multimorbidity. Women had concomitant multimorbidity and disability (18.8%) compared with men (15.5%), while the proportion of inpatients from the south with concomitant multimorbidity and disability (18.8%) and only with disability (5.8%) was greater than that documented in the north (concomitant disability and multimorbidity rate 12.2%, disability rate 1.5%). With increasing age, the disability-only rate increased from 2.2% to 5.6% while the rate of concomitant disability and multimorbidity increased from 2.7% to 33.9%. The prevalence of multimorbidity was highest among those aged 70–74 years (61.4%) (Table 4).

**Table 4:** Characteristics of disability and multimorbidity in older inpatients

**Characteristic**	**n**	**Normal**		**Disability**		**Multimorbidity**		**Disability+ multimorbidity**			
		**n**	**%**	**n**	**%**	**n**	**%**	**n**	**%**	**χ2**	**p**
Total	4348	1193	27.4	144	3.3	2338	53.8	673	15.5		
Sex											
Male	2721	680	57.0	88	61.5	1524	65.2	420	62.4	22.442	<0.001
Female	1636	513	43.0	55	38.5	815	34.8	253	37.6		
Region											
South	1851	523	43.8	106	74.1	854	36.5	368	54.7	134.522	<0.001
North	2497	670	56.2	37	25.9	1485	63.5	305	45.3		
Age											
60–64	777	363	30.4	17	11.9	376	16.1	21	3.1	694.272	<0.001
65–69	766	282	23.6	20	14.0	412	17.6	52	7.7		
70–74	804	221	18.5	19	13.3	494	21.1	70	10.4		
75–79	804	174	12.8	20	14.0	486	20.8	124	18.4		
80+	1197	153	12.8	67	46.9	571	24.4	406	60.3		

Compared to different gender, region, and age group. p<0.05 was statistically significant within the group.

## Discussion

This is a multicenter study of the prevalence of multimorbidity among geriatric inpatients from a representative sample in China. We found that the great majority of older inpatients suffered from chronic diseases, with morbidity and multimorbidity reaching 69.3%. The prevalence of multimorbidity varies by sex, age, and area, as well as by lifestyle and education level. People who suffered two chronic diseases account for the largest proportion. The most common chronic disease among hospitalized older adults is hypertension. In addition to increasing age, living in northern China, living in rural areas, having a low BMI, and less daily activity were independent risk factors associated with multimorbidity.

Age was the most frequently studied determinant of multimorbidity (21–24). We found the incidence of multimorbidity to increase with age. In our study, the prevalence of multimorbidity was 60.7% at ages 60–74 years, 75.7% at 75–79 years, and 81.6% at 80 years or older. These results fit well with the range of the overall prevalence of multimorbidity from studies in China (25) and other countries (5,26–28). A recent systematic review showed that the prevalence of multimorbidity is higher than 60% worldwide, and is probably greater than 80% among those aged ≥85 years (26). Reported prevalence of community-dwelling older adults in Naijing (49.4%) and Guangdong (45.5%) provinces is lower than that in our study (13,29). The rate of multimorbidity in subjects older than 80 years in our research reached 81.6%, similar to that reported by Liu’s group (93.73%). Age is one of the most important factors in organ degeneration and diseases, with the aging process leading to gradual degeneration of organ function to a pathological extent.

A large body of cross-sectional studies conducted in Western countries has examined the epidemiology of multimorbidity (30). In other reviews, the prevalence rates of multimorbidity for inpatients ≥65 years old (61.0%) and those admitted from hospital/healthcare facilities (80.0%) is far higher than in most Chinese domestic studies (31). The prevalence of multimorbidity of all residents of Olmsted County, USA, for example, reached 77.3% at age 65 years and older, possibly related to the higher detection rate of chronic diseases in countries other than China (22). The prevalence of multimorbidity varies widely in the literature because different data sources (questionnaires, medical records, and administrative data) and dissimilar patient populations create differing results. Most estimates are derived from primary care, which relies on participants’ records as the data source. In the present study, we used the diagnoses documented in the medical records, which seems to be a better way of estimating the prevalence of multimorbidity and may produce higher estimates.

In addition, many studies have assessed the association between prevalence of multimorbidity and sex (6,21,32–34). The morbidity rate in men in our study was higher than that in women, contrary to popular belief. Our result is similar to that of the Swiss Family Medicine ICPC Research using Electronic Medical Records (FIRE) (28,33), although other research found that prevalence was significantly higher in women (21,34,35). Our result may be explained by the fact that the sample sources are mostly the cadre wards of hospitals, which mainly contain males; another possible reason may be related to the greater social pressure exerted on men, which may also contribute to the higher mortality rate among older males in China in comparison with female counterparts (36,37).

Our study showed that the multimorbidity rate of older inpatients was higher among people living in northern areas of China than in the south. Surveys conducted in northern China reported a multimorbidity rate in older adults from 82% to 86.9%, compared with 41.8% to 49.4% in southern China (13,38–40). This result could be due to differences in environmental factors and the fact that the lifestyles of older adults in southern China appear to be better than in the north, with people living in southern China having a lower BMI and sodium intake (41). In addition, lower level of education, living alone, fewer daily activities, and abnormal BMI were associated with a higher incidence of multimorbidity (42–44). Our findings were consistent with these previous results.

In the few studies that have addressed risk factors of multimorbidity in China, age, abnormal BMI, and daily activity time were independent factors significantly associated with multimorbidity in older adult inpatients, consistent with our current results (14,42,45,46). In contrast to the findings from Western countries, our study did not find income level to be an independent risk factor for multimorbidity (Table 4), consistent with a large cross-sectional survey of southern China (30,31,47). We also demonstrated that region and area were independent risk factors for multimorbidity. To date, there have been no domestic studies of the influence of residence in the southern and northern regions on multimorbidity. Sex, marital status, smoking, and alcohol consumption were also not found to be independently associated with multimorbidity in the present analysis.

The prevalence of no disease, 1 condition, and 2 conditions were similar in men and women, but the prevalence of more than 2 conditions was higher in men. This result is similar to that reported by Walter’s group, and may be explained by a higher risk of developing more complex combinations of multimorbidity in men than in women, or by an improved survival in men with more complex multimorbidity (22). The results were also similar regardless of whether the inpatients came from northern or southern China.

We found the principal 5 chronic diseases to be hypertension, coronary heart disease, diabetes, cataract, and stroke, whereby hypertension was the single most prevalent disease; therefore, many scholars focus on the study of hypertension combined with other chronic diseases (21). A survey of inpatients older than 80 years from WuHan, China showed that the top 5 common diseases were hypertension (72.51%), cerebrovascular disease (52.29%), coronary heart disease (37.25%), cardiac insufficiency (36.25%), and bone and joint disease (33.96%). Another cross-sectional survey of 4,833 consenting adults aged ≥60 years conducted in 2017 showed that the overall prevalence of hypertension, diabetes, stroke, chronic obstructive pulmonary disease, and coronary heart disease was 50.6%, 10.2%, 6.4%, 5.4%, and 5.5%, respectively, while that of multimorbidity was 16.1% (48). Hypertension affects 1 billion people worldwide and is directly responsible for more than 10 million deaths per year, to the extent that it has been declared a global public health crisis by the World Health Organization (49). In previous years, researchers and medical practitioners have made a tremendous effort to study the comorbidities of hypertension (50,51); specifically, heart disease, diabetes, and obesity are the most widely studied comorbidities (52–56). Therefore, our results are basically consistent with the overall spectrum of chronic diseases.

Multimorbidity impairs the daily life of older people, leading to increased disability rates, frailty rates, and healthcare costs (11). Some research reports weak to moderate associations among geriatric syndromes, multimorbidity, and disability (56). We found that subjects with 2 or more chronic diseases had a higher risk of losing functional independence, as reported in previous literature (57,58). In our study, the disability rates of 18.8% and 15.5% in men and women, respectively, for concomitant comorbidity and disability were higher than results from our previous study of CCGAS (7.0%), but similar to those of a cross-sectional study conducted in community-dwelling older adults aged 60 and older in 12 Hong Kong districts (22.5%) (59). Another study reveals similar prevalence rates of frailty (10.6%), multimorbidity (46.3%), and disability (25.0%) among older adults in Iceland (56). In our study, overlapping prevalence of the two conditions was 15.5%, the higher rate being related to the size of the hospital samples. The prevalence of disability and multimorbidity in women was lower than that in men. The rate of disability in the northern region was higher than in the southern region, but the overlapping rate of multimorbidity and disability in the south was higher than that in the north. This might be attributed to the presence and severity of multimorbidity.

### Limitations and strengths

This study was a multicenter study of multimorbidity of older inpatients across China. Currently there are few studies of multimorbidity among the hospitalized older population in mainland China, and we found that the prevalence of multimorbidity was higher in the north region than in the south region of China, but the proportion of inpatients from the south with concomitant multimorbidity and disability was greater than that documented in the north.

This study has some limitations. First, it is not representative of all older inpatients in China. The actual prevalence of multimorbidity of inpatients might be higher than in our study because there are numerous chronic diseases that we have not covered. Being a cross-sectional study, it cannot demonstrate the relationship between multimorbidity and prognosis, for which a follow-up study is needed. Second, we have only included some of the common chronic diseases; further studies analyzing a wider disease spectrum are warranted.

## Conclusion

China is the largest developing country and also the most populated country in the world. In 2015 the number of people aged 60 years and older was 17% of the population, and this proportion is projected to reach 45% of China’s 1.4 billion residents by 2030 [55, 56]. The aging problem is particularly important in China. Multimorbidity among older people has a high incidence, leading to a decline in quality of life and increased mortality. Multimorbidity is significantly higher in men than in women, and higher in northern than in southern China. Age, abnormal BMI, area, region, and daily activity time are independent factors significantly associated with multimorbidity in older adult inpatients. Alongside the increase in multimorbidity, the health burden and cost are rising. Interventions that suit patients with a single disease may not be appropriate for patients with multimorbidity, and the international guidelines may not be appropriate for Chinese patients. We need to develop more policies to face the threat of aging and multimorbidity.
